# Deepfake Technology and Gender-Based Violence: A Scoping Review

**DOI:** 10.1177/15248380251384271

**Published:** 2025-11-21

**Authors:** Lisa Lazard, Rose Capdevila, Emma L Turley, Kathryn Gilfoyle, Nelli Stavropoulou

**Affiliations:** 1 The Open University, Milton Keynes, UK; 2 Central Queensland University, Brisbane, Australia

**Keywords:** deepfake, online gender-based violence, technology-facilitated sexual abuse, online perpetration, image-based sexual abuse, scoping review

## Abstract

Online violence against women (OVAW) is a growing global problem with deepfakes in gender-based violence as one manifestation of this that has recently attracted considerable attention. This scoping review aims to explore emerging complexities in current academic understandings of deepfake in relation to its use in gender-based violence. The review considers how these issues impact and shape what is currently known about deepfakes in relation to OVAW. Articles were collected between July and September 2024 and then filtered drawing on the Preferred Reporting Items for Systematic Reviews and Meta-Analyses extension for Scoping Review guidelines. Six research databases were searched using 12 search terms compiled by three of the article authors. This resulted in a total number of 3148 articles that were filtered to identify 397 articles that were reviewed in full. The subset was further filtered in order to focus on psychology and the social sciences resulting in a total of 64 articles for analysis. As psychology and the social sciences begin to capture the implications of deepfake creation and dissemination, in the context of online sexual violence, there is a need to investigate how deepfakes are used to silence women in public spaces online, as well as empirically acknowledging the inherent gendered systemic discriminations within deepfake technology and its uses. While important, research must move beyond perceived credibility and detection techniques of deepfakes and toward an analysis of intersectional power dynamics at play in this form of gender-based violence.

## Introduction

Online violence against women (OVAW) is a growing global problem (Benítez-Hidalgo et al., 2024; Jurasz, 2024). A subset of technology facilitated gender-based violence, OVAW exclusively examines problematic online practices that specifically target women, and thus, we prefer this term for the purposes of this article. One manifestation of OVAW that has recently attracted considerable attention and concern is the use of deepfakes in gender-based violence. Coined by a Reddit user in 2017 (Winter & Salter, 2020), the term combines deep learning artificial intelligence (AI) technology and the production of “fake” content. Importantly, the emergence of the term was based on popular descriptors for the creation of non-consensual synthetic celebrity pornographic videos using AI. However, the ways in which deepfakes are defined in the everyday do not always capture the use of deep-learning technology to accomplish such synthetic productions. In academic scholarship, the role of deep learning generative AI has been captured in some definitions of deepfake, but this is not consistently the case (Mumford et al., 2023). This scoping review aims to explore emerging conceptual complexities in current academic understandings of deepfake in relation to its use in gender-based violence. The review considers how these issues impact and shape what is currently known about deepfakes in relation to OVAW. To foreground the gendered nature of non-consensual sexually explicit deepfake abuse, this review will use the term “deepfake gender-based violence” rather than the commonly used phrase “deepfake pornography” except where this term has been used by authors in their studies. A number of factors informed this choice, among which is the recognition that the term “pornography” as a descriptor for abusive material has become increasingly problematized. While “deepfake pornography” remains a common term in both public and academic discourses, the term “pornography” traditionally implies consensual arousal material, whereas the non-consensual deepfake content, which is the focus of this review, constitutes abusive material. This shift mirrors the evolving terminology from “child pornography” to “child sexual abuse material,” emphasizing the non-consensual and abusive nature of such content (NSPCC, 2023) Therefore, in line with these arguments, we acknowledge the need to problematize and reconsider the use of “pornography” in this context to accurately reflect the abusive and non-consensual aspects of deepfake gender based-violence material.

Scholarship on deepfake more generally, and as a form of OVAW specifically, is in its infancy. While, as we have noted, definitions of what constitute deepfakes appear to vary, the etymology of the term is rooted in the idea that deepfake content is synthetically produced by deep learning generative AI. A key characteristic of deepfakes then is the use of specific AI to accomplish digital manipulations that create realistic but entirely false portrayals of sexualized images, videos, and/or audio (Henry et al., 2020). Godulla et al. (2021) have described deepfakes as including: (a) Face replacement or swapping (transfer of one face onto another), (b) Face re-enactment (facial feature alteration), (c) Face generation (creation of faces that are unreflective of real people), and (d) Speech synthesis (creation of voice mimicry).

A recent study reported that 98% of deepfakes are pornographic in nature, 99% of which target women, and that as of 2023, there are 550% more deepfake videos online than in 2019 (Security Hero, 2023). This report underscores the use of deepfake as a form of sexual violence. Within the field of gender-based violence, deepfake has been studied using an array of different terminologies which include or exclude specific aspects, or prioritize distinct elements of deepfakes such as the technique, the use of images, video, or audio and whether these images are “real,” manipulated or created. For the purposes of this paper, we will discuss the use of deepfake in the context of image-based sexual abuse (IBSA) (Henry & Beard, 2024) and technology-facilitated sexual violence (TFSV) (Henry & Powell, 2018). These two catch-all terms are commonly used in the literature to describe a range of violence, abuse, and harassment, directed primarily against women, girls, and minoritized groups online (see Maddocks, 2018; Martínez-Bacaicoa et al., 2024). TFSV refers to a set of behaviors where digital technology is used to facilitate sexual violence in online and offline spaces and encompasses a broad range of harmful behaviors perpetrated via the use of technology (Henry & Powell, 2016). A subcategory of TFSV, IBSA, specifically refers to the non-consensual creation, distribution of (or threats to distribute) or unsolicited sharing of nude or sexualized imagery, and the term recognizes that harms are situated along a continuum of sexual abuses (Henry & Beard, 2024; McGlynn et al., 2017, 2021). Scholarship on IBSA has begun to identify patterns of victimization. More specifically, patterns show that non-consensual gender-based violence disproportionately affects women, including LGBTQ+ women, who are often targeted for online harassment and exploitation more generally (Flynn et al., 2022; Henry & Powell, 2016; Powell & Henry, 2016). This type of content is a serious invasion of privacy and can have severe emotional, psychological, economic, and social consequences for those targeted (Eaton & McGlynn, 2020). Studies of deepfake gender-based violence in the context of IBSA suggest that social and emotional impacts of deepfake gender-based violence on victim/survivors can include reputational harm, relationship difficulties, and physical and mental health challenges such as anxiety and depression (Huber, 2023a). Alongside adult victimization, research on deepfake gender-based violence has also highlighted the use of deepfake technology in promoting pedophilia and producing child-sexual abuse material (Eelmaa, 2022; Internet Matters, 2024). While some studies have examined perpetration dynamics of IBSA (e.g., McGlynn et al., 2021; Umbach et al. 2024), there has been less attention on research that specifically focuses on deepfake gender-based violence, although there is likely to be resonances between different forms of enactment of IBSA and TFSV (e.g., Harder, 2023). Recent scholarship generally focuses on the uses of deepfake as a form of victimization, however, a recent systematic review of deepfakes found that currently, we still know very little about deepfake gender-based violence beyond the initial explorations of prevalence and some aspects of victim experience (Birrer & Just, 2024).

A number of reviews of IBSA featuring deepfakes have been conducted across disciplines such as computer science and education to understand the processes and impact of this technology (e.g., Roe et al., 2024). Such reviews have also included existing research in the broader field of social sciences (e.g., Birrer & Just, 2024; Henry & Beard, 2024; Misirlis & Munawar, 2023). However, the wider focus on IBSA or TFSV in many of these reviews means that there is limited specific focus on deepfakes as a particular and distinct form of gender-based violence. While Birrer and Just’s (2024) review does focus on deepfakes, there is no specific attention to the uses of deepfakes as a form of gender-based violence beyond a concern with “deepfake pornography” as a subset of deepfake misuse. We would argue that a review is needed that considers and explicitly delineates the potential of deepfake research for understanding deepfake gender-based violence.

Importantly, existing articles that review the broader social science literature on deepfakes may forefront computer science research. This is because this field has a comparably larger body of relevant articles that are more specifically focused on deepfakes, compared to social sciences. While this is useful to gain an overall sense of knowledge and understanding of this topic, an unintended effect of this differential disciplinary distribution of research is that contributions from other disciplines such as psychology, criminology, and feminist scholarship may be, at least, partially obscured in these broader reviews. Given the long history of contribution of these disciplines to our understanding of sexual and gender-based violence (e.g., Gavey, 2018; hooks, 2000; Kelly, 1988; Stanko, 1985), we would argue that a review that forefronts these fields is much-needed in order for a comprehensive picture of current empirically based knowledge in the area to be gleaned.

In this scoping review, we bring together studies across psychology, criminology, sociology, feminist scholarship, and related disciplines to both capture what is currently known about this issue as well as identify methodological issues and gaps in need of further research. To start, this review collates varying definitions and conceptualizations of non-consensual deepfake gender-based violence that address the nuances of this topic. The standardization of terminology across IBSA studies is critical to the development of cohesive policies, ethical research frameworks, and in outlining directions for further research. The review will also detail the research methods employed across studies in the social sciences, identifying commonality in methodologies as well as underused, but potentially useful, approaches to this research. As such, this scoping review provides a comprehensive map of the current research on, or which has implications for, non-consensual deepfake gender-based violence.

### Aims and Research Questions

The primary aim was to identify, synthesize, and thematize studies on, or related to, the use of deep fake technology in gender-based violence within specific fields of social science research, with a focus on psychology, criminology, feminist scholarship, and related disciplines. Research questions that guided the process were as follows:

1. How is deepfake gender-based violence defined and understood?
2. What is known about perpetration using deepfake gender-based violence?

## Scoping Review Methodology

Articles were collected between July and September 2024 and then filtered drawing on the Preferred Reporting Items for Systematic Reviews and Meta-Analyses extension for Scoping Review (PRISMA-ScR) guidelines (Page et al., 2021; Tricco et al., 2018). The first phase of the search protocol attended to terminology and definitional issues. Given that deepfake pornography is a common, if not problematic, term used for deepfake gender-based violence, this review began with a search of the terms “Deepfake pornography,” and “deepfake pornography psychology” as well as using keywords “deepfake*” and “deep fake*” on Google Scholar to broadly and initially assess for differing terminology related to this form of violence. Through the review of this literature, other terms were then included such as “non-consensual synthetic intimate imagery” and “non-consensual intimate deepfakes.” Further terminologies relating to, but not necessarily forefronting, deepfake gender-based violence were similarly identified. This iterative process enabled a capture of related terms in the field. A list of 12 search terms were compiled by three of the article authors for a more developed and comprehensive search (see Table 1). We searched each of these terms in six research databases including Google Scholar, PsychArticles (EbscoHost), PsychBooks (EbscoHost), PsychInfo (EbscoHost), Scopus, and Science Direct. The criteria included empirical and theoretical sources that were complete, in English, and published between 2017 (when deepfake as a term was coined) and 2024. Zotero was used as the reference management system for this scoping review.

**Table 1.** Search Term Results.

Search terms	Google Scholar	EbscoHost	Science Direct	Scopus
“deepfake”	138	58	136	1176
“deepfake pornography”	22	3	11	138
“deepfake gender-based violence”	0	13	25	0
“deepfake harassment”	0	1	22	0
“digitised violence”	0	2	65	1
“image-based sexual abuse” (IBSA)	194	85	48	709
“technology-facilitated sexual violence” (TFSV)	136	41	27	646
“non-consensual pornography”	48	23	16	146
“deepfake image based abuse”	0	20	37	0
“non-consensual synthetic intimate imagery”	0	541	1	0
“non-consensual intimate deepfakes”	0	8	1	1
“Cyber sexual violence/harassment”	51	11	5	50
Total	589		394	2867
Overall total				4656

*Note*. • Google Scholar sources used: Psychology; Sociology; Social Sciences; Feminism; Gender Studies; Women’s Studies; and Criminology.

• EbscoHost searches used SmartSearch of key terms.

• ScienceDirect included search based on isolated terms.

As aforementioned, the search was subsequently focused on psychology, criminology, sociology, feminism, and related disciplines, such as gender studies and women’s studies. Despite technical articles and legal literature being excluded as criteria in the search, particular articles based within these disciplines were included due to their multidisciplinary relevance within the broader social sciences. This literature is included within this review, however, with less elaboration than psychology and other social science articles, which are the focus of this report.

### Literature Filtering Processes (PRISMA-ScR Procedure)

The filtering and reviewing process was completed by two of the authors. Disagreements regarding inclusion in the review were resolved by the reviewers by revisiting the inclusion criteria and the screening process to reach a consensus decision through robust discussion. If a consensus was not reached, a third reviewer from the author team was consulted.

The first capture of articles was reviewed, and duplicates were merged and removed, resulting in a total number of *3148 articles*. The literature was then filtered based on titles and abstracts relevant to deepfakes. For example, sources which included the word “deepfake” explicitly, or related terminology exhibiting identical behavior, for example, synthetic imagery, face forgery/manipulation, General Adversarial Networks (GANs), deep learning, fake pornography to name a few. For any articles where the conceptualization of IBSA or TFSV, and whether this included deepfake images, was unclear, the full article was found and read for the terms “deepfake” or “deepfakes” to determine article relevance. Any full articles that were not found to be in the English language (e.g., mistakenly picked up in the review) were excluded. Forty-one articles were initially appraised as unclear as to their relevance to deepfake gender-based violence. These articles were read in full and assessed against the inclusion criteria. This process resulted in 6 out of the 41 articles being retained for inclusion.

In common with other scoping reviews (e.g., Birrer & Just, 2024; Henry & Beard, 2024), the filtered results were compared and checked against reference lists within full research articles, and other scoping reviews on deepfakes. This was done to capture further relevant deepfake literature that may not have been picked up through the search terms. These articles were also added so that this review should remain a comprehensive representation of the literature surrounding deepfakes. The filtering process resulted in a total of 397 articles that were reviewed in full (Figure 1).

**Figure 1.**
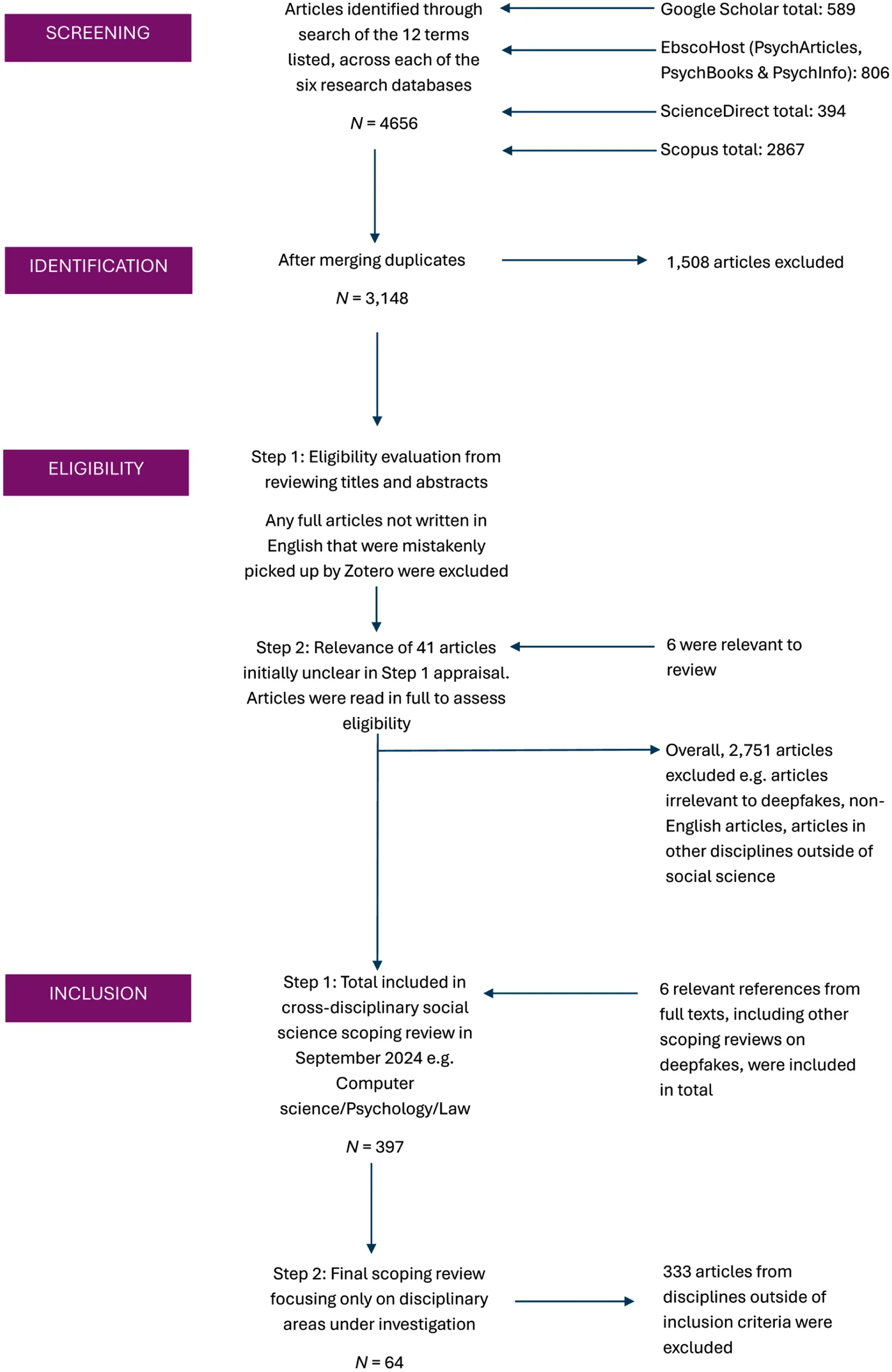
PRISMA diagram.

The subset of 397 articles was further filtered in order to focus on the disciplinary areas under review and to remove computer science as per the rationale described above. Legal scholarship was excluded at this stage, as our research questions focused on definition and perpetration rather than regulation. Given that everyday sense-making of deepfakes has been identified as posing a challenge for the implementation of regulation (McGlynn et al., 2024), we argue that our questions must first be addressed through the disciplines covered in this review. This review can thus serve as the precursor for further examination of legal approaches, building on the groundwork established here.

Once completed, the filtering process resulted in 64 articles for analysis. The articles were coded and organized thematically to allow for comparison of study focus, methods, and theoretical and methodological limitations. However, we retain mention of some disciplinary boundaries where appropriate in the following reporting to highlight specificities in respective areas. The thematic approach to article review was used to identify underused, but potentially useful, methodological approaches to researching deepfake gender-based violence.

## Results

With a focus on the research questions detailed earlier, the review identified several key themes. These included challenges related to terminology, the prevalence, and various types of deepfake, and insights into the roles of perpetrators, victims/survivors, and consumers of this material (Tables 2 and 3).

**Table 2.** Critical Findings.

Terminology	Deepfake gender-based violence is often integrated within umbrella terms such as technology-facilitated sexual violence (TSFV) or image-based sexual abuse (IBSA) or conflated with other digitally altering behavior such as photoshopping. This lack of specificity in terminology hinders research defining and studying deepfake gender-based violence as a distinct behavior.
Prevalence	Due to existing terminology issues, there is difficulty in reporting the prevalence of deepfake gender-based violence specifically. Further research that exclusively focuses on non-consensual deepfakes is needed to accurately investigate prevalence.
Demographic risk factors	Research suggests that perpetration of deepfake gender-based violence is primarily boys and men, with young social media users perpetuating further sharing of deepfaked content. Statistical findings suggest non-consensual sexualized deepfakes are disproportionately used to target women, including public figures and politicians, positioning deepfake victimization as a form of gender-based violence against women, girls, and minoritized groups.
Nature and scope of offending	Deepfaked content is primarily pornographic/sexualized and targeted toward women and girls. It therefore should be considered a form of online gendered violence underpinned by psychological motivations for other forms of sexual offending, revenge and control, community engagement, and cultural displays of hegemonic masculinity.
Impacts and harms	Being a victim of deepfake gender-based violence causes social and emotional impacts such as reputational harm, relationship difficulties, and physical and mental health challenges, such as anxiety and depression. However, scant research has explored the deepfake victim experience.
Reporting and help-seeking	There is limited knowledge on how victims respond to deepfake gender-based violence, as similar to other forms of TSFV and IBSA, victims are often stigmatized and blamed. Research has suggested women adopt self-protective behaviors online to prevent victimization, and there is a discrepancy between perceived self-efficacy and accuracy in detecting deepfake content, both of which could influence reporting of deepfake victimization.

**Table 3.** Recommendations for Research, Policy, and Practice.

Research	Policy	Practice and Engagement
• A precise definition of deepfake gender-based violence is necessary for future study, distinguished from other forms of image manipulation such as photoshopping, and distinct from generalized behaviors included within TSFV and IBSA. • Greater inclusion of feminist frameworks in academic research and policy production, investigating deepfake gender-based violence within the broader landscape of gender inequality, and the devaluing women’s agency		
• An intersectional research approach to examine how race and ethnicity, sexuality, disability, and class integrate with gender to inform victim/survivor’s experiences of deepfake gender-based violence. • Investigations into the use of deepfakes as a silencing tool toward women in high-profile, political positions, outspoken feminists, or LGBTQ+ minorities. • Explorative research examining the facilitation of intersectional inequalities within deepfake algorithms, machine learning, and content creation. • Comprehensive qualitative and quantitative research is needed examining the prevalence, risk factors, and demographic information of deepfake gender-based violence victimization–perpetration to reflect current rates of all forms of this behavior (image, video, audio) intersectionally and cross-culturally. • A comprehensive analysis of deepfake gender-based violence within online communities, exploring the socio-cultural factors which perpetuate the creation, discussion, and consumption of non-consensual deepfake imagery.	• Awareness-raising campaigns to highlight the legal, social, and civil implications of creation and distribution of deepfake gender-based violence. • Clear and consistent training on the impacts of deepfake gender-based violence and best practice responses for law enforcement and the court system, schools, and youth organizations. • Increased collaboration between various academic organizations, NGOs, technology companies, and government agencies to understand and respond to deepfake gender-based violence.	• Educational initiatives that centralize the idea of sexualized and gendered deepfake creation as a form of gender-based violence. • Educational interventions with young people in relation to deepfake creation, further sharing of content, and attributions of responsibility for bystander behavior. • Targeted educational interventions to highlight the discrepancy between detection self-efficacy and accuracy, raising awareness of false online content, especially in a gendered context.

### Research Question 1: How Is Deepfake Gender-Based Violence Defined and Understood?

#### Terminology

The review indicated that deepfakes as a form of gendered violence have only recently been investigated in their own right, likely because they were not publicly available until 2017 and, more recently, due to the frequent integration of deepfake abuse with other forms of IBSA or TFSV. In reviews of TFSV, deepfakes are defined, and often included, as part of IBSA (Monteiro et al., 2024), featuring alongside non-consensual distribution of intimate images and sextortion (Henry et al., 2020). For example, deepfake gender-based violence has frequently been conflated with other types of IBSA such as photoshopped sexualized images (Huber, 2023a). There is a significant difference between photoshopped, and deepfake images. Deepfakes are technologically sophisticated products that look or sound more realistic than their photoshopped counterparts. Importantly, it is very difficult, and sometimes impossible, to prove deepfakes are indeed fake (Köbis et al., 2021; Korshunov & Marcel, 2020). This is not the case for photoshopped images, which can be shown to have been manipulated. This conflation of definitions was evident in Mumford et al.’s (2023) study examining the prevalence of victimization by TFSV in a young adult population in the United States (*N* = 2,676, aged 18–35), in which “digitally altered images” as a victimization item was operationalized as both photoshopped images and deepfakes.

There have been attempts to conceptually distinguish deepfake from other forms of IBSA. For example, Harper et al. (2021), in their paper on delineating non-consensual sexual image offending, distinguished deepfake media production from other forms of non-consensual sexual images, such as those known by the problematic term “revenge porn.” Similarly, Popova’s (2020) investigation of pornographic deepfakes of celebrities pointed to the differences between deepfake videos and other forms of digital blending of celebrity images. However, concomitantly, Paradiso et al.’s (2024) systematic review, which aimed to examine victimization and propensity to perpetrate IBSA, included deepfakes as a form of this behavior.

Overall, there remains a limited focus specifically on deepfake gender-based violence. There is, however, acknowledgement that it is an area requiring further research and one that could be a fruitful future direction for research into IBSA (e.g., Harper et al., 2023; Maddocks, 2018; Martínez-Bacaicoa et al., 2024; Plohl et al., 2024). We would argue that the falsified nature of deepfakes, along with the difficulties of evidencing their falseness, positions this form of IBSA differently to others. This is particularly the case in relation to the experiences of and recourses available to perpetrators, victim/survivors, and consumers as well as legislators and policy makers. This broad application of the term can also undermine the distinctiveness of deepfakes, which require complex machine learning algorithms to produce, and which pose unique ethical, legal, privacy, and security challenges compared to simpler forms of image editing. This misapplication of terminology may confound research efforts, as it fails to capture the specific risks and consequences associated with deepfake technology and AI. For these reasons, we would further propose that future projects should aim to design research that positions deepfakes, as opposed to fake content, as the object of focus, and explicitly explore deepfake gender-based violence.

#### Prevalence

Currently, research on prevalence as well as patterns that characterize deepfake victimization and perpetration is scant. Examinations of deepfake gender-based violence victimization are often investigated through a legal or regulatory lens, rather than from psychological or criminological ones (see, for instance, Umbach et al., 2024). This, as mentioned in the previous section, is further complicated by the conflation of deepfake gender-based violence with other forms of IBSA, particularly “revenge pornography,” when assessing prevalence statistics. Bansal et al. (2024) have argued that, given the existing conflations, it is difficult to establish the prevalence statistics of deepfakes specifically, while a scoping review of IBSA by Henry and Beard (2024) found no prevalence statistics for the perpetration of creating deepfake images, as these were embedded in the statistics for IBSA. This is further evidenced in Turner et al.’s (2025) research on prevalence and characteristics of perpetration and victimization in IBSA in the USA. While deepfakes were referenced as a form of image-based sexual exploitation alongside photoshopping, these different forms were included together under a more general focus on the perpetration of non-consensual image sharing. This pattern is visible in Sanders et al.’s (2023) U.K. mixed-method study on IBSA experiences of commercial creators whose content has been misused. In Sander et al.’s study, deepfakes were included within the definition used; however, due to conflation with other forms of IBSA, there was a lack of study of the specificity of deepfake victimization. While a recent review by Sheikh and Rogers (2024) on TFSV research claimed that 6% of women in South Asian countries experienced deepfake harassment, the statistic arose from a conflation of deepfakes with “synthetic porn,” which included photoshopped pornographic images. Deepfake gender-based violence was not specifically distinguished. Indeed, the relationship between impersonation and deepfake in the above study appears to be derived from an article from a media and entertainment company (Ruíz et al., 2020). Thus, the literature indicates that there is a propensity to mention or acknowledge deepfake as a form of image-related gender-based violence without a specific focus on it as a topic of study in its own right.

This lack of deepfake specificity also extended to cross-cultural research. Rackley et al.’s (2021) 3-year cross-jurisdictional project with quantitative surveys and qualitative interviews across the United Kingdom, New Zealand, and Australia assessed the prevalence, nature, and impact of IBSA. In this study, deepfake was mentioned alongside other forms of IBSA (e.g., fakeporn and Photoshop), however, excerpts from interviews with 75 women on their experiences of image manipulation, discuss Photoshop rather than the use of deepfake technology *per se*. While it is certainly possible that Photoshop and deepfakes are used interchangeably in everyday discourse, the lack of clarifications means that it is not yet possible to separate findings specifically about deepfake from other forms of IBSA.

Exceptions to the above pattern, which have specifically focused on deepfakes, include a recent doctoral thesis (Bahrenburg, 2024) located in psychology, which examined demographics of people victimized by different types of cyber sexual abuse (*N* = 126, 82.5% aged 18–26). This research found that victimization through deepfake pornography was the least common form of abuse (3 participants, 2.4%). Data collection materials for this study, however, defined deepfake as an “image superimposed on a sexually explicit video or image to appear as if you are acting out the sexually explicit material” (p. 82), so as including all forms of image manipulation. Therefore, it is likely that participants may have reported other forms of IBSA instead of, or alongside, deepfakes specifically because of the conflation of terms in everyday sense-making as argued above.

Taken together, this review suggests that there is a considerable gap in current knowledge on the prevalence of victimization–perpetration as well as methodological procedures that enable respondents to distinguish deepfake gender-based violence from other forms of IBSA.

### Research Question 2: What Is Known About Perpetration Using Deepfake Gender-Based Violence?

#### Social Perceptions and Treatment of Deepfake: Sharing and Responsibility

Social media has been identified as playing a key role in public exposure to non-sexual deepfakes, and the often-comical nature of the deepfake content shared on these sites appears to redirect responsibility away from online platforms. Indeed, there is some suggestion that the media has contributed to the promotion of deepfake gender-based violence as less important than deepfakes used for disinformation (Gosse & Burkell, 2020). However, a study that examined online audience responses to witnessing “deepfake pornography” found that while audience engagement varied, anger was more strongly associated with bystander intervention than guilt (Wang & Kim, 2022). One survey found that awareness of deepfakes is determined by intensity and type of social media use, and that the level of humor of the deepfake content can negatively affect perceived responsibility of platforms to regulate deepfakes (Cochran & Napshin, 2021). There is a lack of confidence in technology being able to solve the problem of deepfakes (Cochran & Napshin, 2021), which attributes responsibility to individuals. Further cyberpsychology research on individual responsibility attributions for creation and sharing of non-sexual deepfakes found that people externalize responsibility for deepfake images. For example, younger people who find deepfakes humorous are more likely to perceive themselves as responsible for sharing this content (Napshin et al., 2024). Such studies may support further research into bystander response to audiencing or sharing deepfakes on social media platforms, including slowing the spread of distribution. This is an important area, with educational implications (Ali et al., 2021).

Alongside responsibility considerations around sharing deepfakes, experimental studies have examined social judgements of sexualized deepfake images. Fido et al. (2022) found that people have more lenient judgments of sexualized deepfakes if the image is of a celebrity, of a man, and if the image was for self-sexual gratification as opposed to being publicly shared. Fido et al.’s study points to the gendered nature of deepfake victimization, the sexual double standards for public figures, and the influence of social perceptions on public sharing of sexual deepfakes. In a similar vein, Maddocks (2020), found, in a sample of 3000 tweets about or on deepfake content, that there was a tendency for this form of gendered violence to be overlooked, marginalized, and automatically distributed by bot accounts. Taken together, these studies point to gendered inequalities not only in patterns in victimization–perpetration but in wider social and cultural responses to deepfake as a form of gendered violence. A recent study by Harper et al. (2023), which developed the Beliefs about Revenge Pornography Questionnaire, suggested that this tool could be developed into an adapted version to examine beliefs about deepfake gender-based violence. However, at the time of this study, there are no known measures of beliefs about deepfake offending.

#### Deepfake Perpetration—Creators and Consumers

Research examining motivation for the creation and consumption of deepfake images is in their infancy. While not explicitly on deepfakes as a form of violence, Marini et al. (2024) conducted a cognitive psychological experiment concerned with the authenticity of content in relation to consumption of “deepfake pornography.” In this experiment, 27 women and 30 men were asked to view real pictures of men and women models wearing lingerie or swimsuits and were told that the images were either of real people or artificially generated. The findings from this experiment suggested that perceived realness of the images was highly positively correlated with participant’s self-reported sexual arousal. While the rationale for this experiment was not explicitly related to questions around motivations of perpetration or consumption of non-consensual deepfake gender-based violence, it may be of relevance to this issue. This is because the findings may point to actual experiences related to, or broader social acceptabilities around, the relationship between deepfake viewing, sexual gratification, and talking about such consumption.

The purposes of deepfake creation have been recognized as a highly complex phenomenon (Tanwar & Mahapatra, 2024). Frameworks for understanding offline sex offending have been applied to online gendered violence, including deepfake creation. In common with Marini et al.’s (2024) conclusions, the idea of sexual gratification as a motivation for deepfake creation was highlighted in Harper et al.’s (2021) review of non-consensual deepfake creation as sex offending. Drawing on the sex offending literature, Harper et al. (2021) argue that motivations for deepfake creation can be linked to the underpinnings of other forms of sex offending, for example, dysfunctional sexual scripts, sexual compulsivity, and self-sexual satisfaction. More generally, Vasist and Krishnan (2023) argued that creators of sexualized deepfakes are often represented in research as malicious in their intent to, for example, enact reputational damage, control, and exact revenge against those depicted in deepfakes. However, Vasist and Krishnan (2023) note that a focus on motivation alone is not a sufficient explanation for deepfake creation. This is because deepfake creation is becoming a highly stigmatized and criminalized practice across various jurisdictions globally. As a result of these burgeoning legislations, open-source methods for creating deepfakes are becoming less accessible. However, the challenges of creating deepfakes have decreased, allowing creators to produce deepfake gender-based violence content with only limited technical expertise.

Moving away from individualized accounts of motivation, there has been a small number of studies that have explored deepfake creation and consumption in online communities. Popova (2020) explored communities that engaged with digitally created sexual content of celebrities, which included deepfake celebrity videos. Popova was particularly interested in the ways in which authenticity and intimacy become relevant to audience engagements that seek to access the “real” person outside of the celebrity image. Popova argues that while some sexualized content such as celebrity hacks are related to accessing something real or authentic in the lives of celebrities, within particular communities’ engagement with digitally altered sexualized images is located within broader practices of fictional imaginaries (akin to fan fiction). Deepfake sexualized content of celebrities is similarly not tied to the lives of the celebrities themselves, according to Popova. Instead, they are tagged as fake, unreal, and artifacts of digital alterations of both celebrity and porn and are intentionally contained within the community rather than distributed more widely, as in the case of celebrity hacks. In these cases, Popova argues, deepfakes appear to be for “relatively private pleasures shared only with those who have a similar understanding of the material” (p. 21).

Communities associated with deepfake creation were also the object of study by Newton and Stanfill (2020) who examined discussions of deepfakes on Github, which is a platform for software development and primarily androcentric. Discussions were located in a broader culture of toxic “geek” masculinity in which technological expertise is harnessed to perform hegemonic masculinity, while at the same time, claiming a marginalized masculine status. The creation and discussion of non-consensual deepfake pornography were accomplished by abstracting creation from the human, disavowal of pornographic elements, and an indifference to harm. However, the social unacceptability of deepfake creation practices was recognized by disguising creator identities. Newton and Stanfill (2020) conclude that these patterned ways of discussion suggest that “deepfakes serve as a site for using technology to enact hegemonic masculinity’s dominance while hiding behind technocratic rationality to refuse empathy” (p. 11). Similar patterns were observed by Winter and Salter (2020) on community interactions on deepfakes on Reddit and Github and are consistent with Kalpokas et al. (2024) review on non-consensual deepfake creation and sharing, which reflect an underlying culture of toxic masculinity. This is also resonant with Ayers’ (2021) work on how deepfake face swaps perpetuate white male hegemony and masculine power in YouTube videos, echoing the androcentric and patriarchal power imbalances located in deepfake creation. Deepfake gender-based violence has thus been conceptualized as a medium through which creators can perform a version of hypermasculine violence and is also concerned with entitlement enacted through the coming together of online communities for sharing non-consensual synthetic sexual imagery (Kalpokas et al., 2024). This entitlement to procure deepfake non-consensual imagery is flagged in Kikerpill (2020) who analyzed a thread on a community site that was dedicated to deepfake “designer pornography” of a person they had a relationship with during secondary school.

The research on online communities identified in our scoping review points to the ways in which creation and consumption are embedded within social practices. This makes particularly relevant Hancock and Bailenson’s (2021) call for future research focusing on social issues around deepfake creation, while keeping updated with evolutions in the capabilities of these technologies. For example, they suggest that this could include investigations of deepfakes used for live video as opposed to pre-recorded video, which is the form of deepfake that is predominantly discussed in current literature.

## Discussion

This review aimed to explore how deepfake gender-based violence is defined and understood, and what is known about perpetration involving this form of abuse against women and girls. The findings illustrated significant conceptual, definitional, and evidence gaps that hinder a comprehensive understanding of deepfake gender-based violence to which we now turn.

### Identifying Deepfakes

In common with Birrer and Just’s (2024) review, the analysis presented here identified a concern with the identification or detection of deepfakes. While research on the topic of deepfake identification has typically noted the importance of this area of study in relation to deepfake misuse, the focus of these studies was not on gender-based violence per se. This area of investigation is characterized largely by experimental studies on face and auditory recognition as well as perceptions of deepfakes, including the perceived credibility and trustworthiness of them (e.g., Bankov et al., 2021; Jin et al., 2023).

Currently, there are mixed findings on the human ability to distinguish between computer-generated and human faces. This includes experimental psychological research indicating some characteristics of AI faces were found to be indistinguishable from those of humans, including aspects of emotion perception (Becker et al., 2024; Becker & Laycock, 2023; Miller, Foo, et al., 2023) and how the faces can be judged as more human (Miller, Steward, et al., 2023). This difficulty in identifying synthetic faces also translates to synthetic voices (Öhman, 2022), suggesting human visual and audio processing may not be able to reliably distinguish between human and technologically created voices. Despite these human limitations, findings suggest that people are more accurate at detecting deepfakes when the person in the image is familiar, when they spend more time on social media, and when they are prone to conspiracy thinking (Nas & de Kleijn, 2024). Other experiments have found that people identify deepfakes using both video and audio cues to interpret the messages conveyed in the video (Goh, 2024; Groh et al., 2022). Despite a positive emphasis on using human detection methods to identify deepfakes, some researchers suggest there can be a third-person perception bias where people perceive themselves as better at discerning deepfakes compared to others, which they attribute to higher cognitive ability. However, this bias has little influence on one’s actual ability to differentiate authentic and deepfake imagery (Ahmed, 2023). Even in people with high cognitive ability, deepfakes without obvious cues are more likely to be considered true and shared (Ahmed, 2021). Indeed, research in assessing people’s perceived self-efficacy in detecting deepfakes has found that people overestimate their pre-emptive abilities to detect deepfakes (Somoray & Miller, 2023). This aligns with other research findings that participants’ confidence in distinguishing deepfake images from human images was high. Confidence was, however, unrelated to the accuracy of detection (Bray et al., 2023).

While the literature is somewhat mixed in terms of people’s deepfake detection skills (Groh et al., 2022), this research emphasizes a contradictory psychological belief in one’s inherent ability to recognize technologically manufactured content. This could have serious implications for victims of deepfake gender-based violence in which real pornographic content is perceptually or interpretatively indistinct from fabricated images or videos (Godulla et al., 2021). However, little research explores human perception and visual processing in a gendered context. There is potential for such research to inform gender-based violence interventions where, for example, detection self-efficacy is compared with their accuracy, to raise awareness of the possibility that online content is not always as real as it appears. Nightingale and Wade (2022) argue that there is a need for research that examines ways to improve people’s detection of subterfuge.

### Understanding Victimization

The review revealed substantial gaps in knowledge about both victimization and perpetration in the context of deepfake gender-based violence. Victimization studies often lack specificity due to the terminology conflations outlined above, making it difficult to discern the specific impacts of deepfake gender-based violence on women and girls compared to other forms of IBSA and TFSV. This scoping review illustrated that studies rarely address the broader socio-cultural contexts that shape how victims experience and respond to this specific type of abuse, which may include shame and stigma. Studies also neglect to examine the intersection of gender with other identities such as race, class, or sexuality (Rice et al., 2019). This limits knowledge relating to women’s and girl’s experiences in terms of similar and differing socio-psychological and traumatic impacts of deepfake gender-based abuse compared to other forms of IBSA and TFSV.

### Overlooking Gender in Deepfake Abuse

Another key finding of this review is the extent to which the gendered nature of deepfake abuse is overlooked in much of the existing research. The conflation of various types of gender-based IBSA with broader categories of technology-facilitated violence, such as doxing and identity theft, obscures the gendered nature of deepfake abuse. While online abuse against women often reflects broader societal patterns of misogyny and gender inequality (Harris & Vitis, 2020), creators and sharers of explicit deepfake content specifically exploit sexualized imagery to enact power and control over women, representing a “new form of digital sexual abuse” (Jacobsen & Simpson, 2023, p. 1099). However, as the review illustrates, this distinctly gendered dimension is often overlooked in academic research, reflecting cultural and social norms that fail to interrogate deepfake content’s intersection with gender-based violence.

The review demonstrates that deepfake abuse disproportionately targets women, including those in public-facing roles such as politicians or celebrities (Maddocks, 2020; Security Hero, 2023). However, discussions of this phenomenon often focus on the technological aspects of deepfake creation, deepfake detection abilities, and the broader implications for misinformation and digital ethics, minimizing the specific ways in which deepfakes can be used to reproduce and perpetuate gendered inequalities in online and offline spaces. Cultural and social norms around “deepfake pornography” further compound this issue. The normalization of non-consensual sexualized content and the broader societal trivialization of harms associated with IBSA or “revenge pornography” contribute to a lack of recognition of deepfake abuse as a form of gender-based violence (Huber, 2023b). The tendency to treat deepfakes as a neutral technology without recognizing the gendered power dynamics involved can obscure how these technologies perpetuate broader patterns of gender inequality (Gross, 2023). This is reflective of deeper societal patterns that devalue women’s autonomy and perpetuate harmful practices around sexuality and consent (Baldwin-White, 2019).

### Social Inequalities, Intersectionality, and Feminism

In the preceding sections, the operation of gendered power inequalities and associated power dynamics has been highlighted as a gap in existing work on deepfake creation. The relevant notion of the male gaze to understand deepfake pornography were noted by Wagner and Blewer (2019) because of the assumed predominance of cisgender, presumably heterosexual men using and consuming images depicting cisgender women. As such, they argued for the need for feminist perspectives on AI and deepfakes, which aligns with other academic positions discussing deepfakes using frames around patriarchal power relations in operation in the manipulation of women’s visual content (Jacobsen & Simpson, 2023).

We would argue that current research indicates the need for an intersectional approach to the analysis of gendered power. For example, as indicated by Sen and Jha’s (2024) study of deepfake abuse in India, deepfake pornification and sexually manipulated images are used to mute women in cyberspace who defy cultural and religious belief systems. There is currently a small number of articles that point to intersectional power dynamics at play in the creation and consumption of deepfakes. These include, for example, experimental research on how deepfake manipulations of physical attractiveness can affect perceptions of teaching ability (Eberl et al., 2022) and how using deepfake technology to alter a video of a Black person to appear whiter increases perceived credibility of the person telling the truth (Haut et al., 2021). These experimental studies, however, are potentially limited in terms of what they can offer for understanding intersecting and often subtle power relationships and discriminations within feminist intersectional frames. Previous research has indicated that facial data in AI is inherently patriarchal and discriminatory against people of color, for example, Snapchat filters lighten skin color when designed to appear more feminine, and deep learning models (GANs) produce non-white women with more masculine features and lighter skin when generating engineering professors (Jain et al., 2022). As such, there are numerous avenues for future research to explore gender-based inequalities that are facilitated by AI and deepfakes. This could include specific attention to how raced and gendered inequalities [such as Bailey’s (2021) work on misogynoir] become relevant in the creation, consumption, and experience of deepfake gender-based violence.

It is also worth noting how the emergence of deepfake gender-based violence material has impacted consensual DIY pornography practices. These practices have been conceptualized as a diverse presentation of online pornographies underpinned by displays of new, alternative, and often marginalized sexual cultures, including queer porn makers and performers (Jacobs, 2024). As Jacobs (2024) suggests, such DIY porn can promote an “ethics of inclusivity” (p. 94), but this has been impacted by deepfake gender-based violence as a form of hate media, which may curtail creation and consumption of consensually created DIY porn, designed in this spirit of inclusivity. This may be particularly the case given the violent targeting of “outspoken feminists and LGBTQ+ minorities, especially those who are public figures from ethnic minority backgrounds” (p. 95). Jacobs (2024) undoubtedly draws attention to the indirect effects of non-consensually created deepfakes as a form of gender-based violence in the context of hate media and the ways it can be used to silence and erase minoritized groups.

How women are responding to deepfakes is an area that is beginning to receive attention. It is important to note that deepfake is not something that is solely *done to* women, and research has highlighted how women’s own use of deepfakes can positively mediate their sense of their own bodies (Wu et al., 2021). That said, non-consensual deepfake objectification can mean that women can lose control and agency over both their images and their bodies (Jacobsen & Simpson, 2023). How women protect against online abuse, including “synthetic porn,” was explored by Sambasivan et al. (2019), which included using images that were not of them (e.g., animals or landscapes) in their online profiles. van der Nagel (2020) explored women’s agency in challenging consent in instances of deepfake. Her case study examined sexual consent in a Reddit forum, which had a verification system to ensure the photos were consensual, underscoring the importance of this in the production and consumption of porn. While women’s engagements with self-protection is a pertinent area of study, we would argue that future research must attend to creators and consumers, as well as victims, in efforts to protect against and prevent deepfake gender-based violence. This serves to mitigate against victim-blaming that has long been commonplace in popular and can also be found in academic, discussions of preventing violence against women and girls.

## Limitations

While we endeavored to capture the breadth of work addressing our research questions, there remain limitations to this study that curtail the claims that can be made and also offer opportunities for further study. A potential issue is the probability that we did not include some search terms that might have enriched the source material, for instance, AI or GenAI, NSII, or NCDII. As a result of the disciplinary focus, which was necessary to the coherence of the review, some relevant studies were missed due to being published in journals that would not have been flagged as relevant to the disciplines under consideration. In addition, this review, as a scoping review, did not engage fully with the specifics of the analyses carried out in the papers analyzed, be they quantitative or qualitative, and so has restricted itself to reporting on these studies rather than conducting a critical analysis. This scoping review may thus provide the basis for a systematic review, including research questions focused on explicating conceptual and methodological approaches used in the study of deepfake gender-based violence. This may be a particularly fruitful avenue to pursue given the challenges of assessing prevalence, and concomitantly, perpetration dynamics and specific impacts of this particular and distinct form of OVAW on victim/survivors. Of note is that currently, the study of victimization–perpetration dynamics has focused on women as victim/survivors and men as perpetrators because this remains the most prevalent manifestation of gender-based violence both on and offline. This review, as reflective of the current state of knowledge, is limited in relation to understanding other forms of victimization/perpetration, particularly the victimization of men. This, as a potential direction in future research, may deepen understanding not only of online gender-based violence but also of contemporary masculinities.

## Future Directions and Concluding Remarks

Overall, this scoping review highlights the need for research into a plethora of academic avenues exploring this area. Given the proliferation of deepfake gender-based violence (Winter & Salter, 2020), prevalence statistics of deepfake pornography in all forms (image, video, audio) are becoming increasingly important in order to adequately appraise the scope of the problem, which is problematized by the difficulty in identifying deepfakes. This should include an exploration of the platforms that circulate this content, the ratio of pornographic to non-pornographic deepfakes, and with attention to victims and perpetrators.

As psychology and the social sciences begin to capture the implications of deepfake creation and dissemination, especially in the context of online sexual violence, academics and researchers must be explicit with their terminology and precise with their definitions. There is the potential to investigate how deepfakes are used to silence women in public spaces online, as well as empirically acknowledging the inherent gendered systemic discriminations within deepfake technology and its uses. While important, research must move beyond perceived credibility and detection techniques of deepfakes and toward an analysis of intersectional power dynamics at play in this form of gender-based violence.

## Supplemental Material

sj-docx-1-tva-10.1177_15248380251384271 – Supplemental material for Deepfake Technology and Gender-Based Violence: A Scoping Review Supplemental material, sj-docx-1-tva-10.1177_15248380251384271 for Deepfake Technology and Gender-Based Violence: A Scoping Review by Lisa Lazard, Rose Capdevila, Emma L Turley, Kathryn Gilfoyle and Nelli Stavropoulou in Trauma, Violence, & Abuse

## Data Availability

The review used publicly available which is referenced in a supplementary document to this article.
