# Supplementary material for: Deepfake Technology and Gender-Based Violence: A Scoping Review
Source: Trauma Violence Abuse. 2025 Nov 21;27(5):2666–81. doi: 10.1177/15248380251384271 (PMC13736260; doi:10.1177/15248380251384271)
Supplement: Supplementary file 1 [file sj-docx-1-tva-10.1177_15248380251384271.docx]

*Table 4. Final set of 64 papers in review*

|  | Paper reference | Discipline | Methodology | Key Findings from review |
| --- | --- | --- | --- | --- |
| 1 | Ahmed, S. (2021). Fooled by the fakes: Cognitive differences in perceived claim accuracy and sharing intention of non-political deepfakes. *Personality and Individual Differences*, *182*. https://doi.org/10.1016/j.paid.2021.111074 | Psychology | Quantitative (Online survey) | People with high cognitive ability are more likely to perceive deepfakes as true, therefore share deepfake content, when informative cues are missing. |
| 2 | Ahmed, S. (2023). Examining public perception and cognitive biases in the presumed influence of deepfakes threat: Empirical evidence of third person perception from three studies. *Asian Journal of Communication*, *33*(3), 308–331. Scopus. https://doi.org/10.1080/01292986.2023.2194886 | Psychology | Quantitative (Online survey) | Third-person perception bias is not predictive of one’s actual ability to distinguish deepfakes from real content. |
| 3 | Ajder, H., Patrini, G., Cavalli, F., & Cullen, L. (2021). *Info@deeptracelabs.com*. | Cybersecurity | Landscape report | 96% of deepfake videos online are pornographic. 94% of deepfake porn is on dedicated websites, and 8 out of top 10 pornography websites host deepfaked content. |
| 4 | Ali, S., DiPaola, D., Lee, I., Sindato, V., Kim, G., Blumofe, R., & Breazeal, C. (2021). Children as creators, thinkers and citizens in an AI-driven future. *Computers and Education: Artificial Intelligence*, *2*. https://doi.org/10.1016/j.caeai.2021.100040 | Computing and Education | Workshop data with school students and questionnaire (Quantitative analysis) | Young people can recognise generative AI is believable yet not real, and participate in spreading disinformation. |
| 5 | Ayers, D. (2021). The limits of transactional identity: Whiteness and embodiment in digital facial replacement. *Convergence*, *27*(4), 1018–1037. Scopus. https://doi.org/10.1177/13548565211027810 | Media communication | Essay | Deepfake face swaps perpetuate white male hegemony and masculine power in YouTube videos. |
| 6 | Bahrenburg, S. E. (2024). *Cyber Sexual Abuse victimization: A mixed methods approach through existential phenomenology* (2024-78562-012; Issues 9-B). ProQuest Information & Learning. | Forensic Psychology | Mixed methods (Quantitative and Qualitative surveys) | Deepfake pornography (all image manipulation) victimisation was the least common form of abuse (3 participants, 2.4%). |
| 7 | Bankov, K. (2021). Face and trust: A semiotic inquiry into influencers, money, and amygdala. *Sign Systems Studies*, *49*(3), 527–542. Scopus. https://doi.org/10.12697/SSS.2021.49.3-4.14 | Semiotics | Literature Review | Explored interplay between semiotics, face and perceived trustworthiness, whereby trustworthiness of the face will play a significant role in synthetic content on social media. |
| 8 | Bansal, V., Rezwan, M., Iyer, M., Leasure, E., Roth, C., Pal, P., & Hinson, L. (2024). A scoping review of technology-facilitated gender-based violence in low- and middle-income countries across Asia. *Trauma, Violence, and Abuse*, *25*(1), 463–475. Scopus. https://doi.org/10.1177/15248380231154614 | Psychology | Scoping Review | Prevalence of deepfake victimisation often conflated with other forms of IBSA such as revenge pornography. |
| 9 | Becker, C., Conduit, R., Chouinard, P. A., & Laycock, R. (2024). Can deepfakes be used to study emotion perception? A comparison of dynamic face stimuli. *Behavior Research Methods*. Scopus. https://doi.org/10.3758/s13428-024-02443-y | Neuroscience | Experimental questionnaire (Quantitative and Qualitative) | AI faces found to be indistinguishable from those of humans, including aspects of emotion perception. |
| 10 | Becker, C., & Laycock, R. (2023). Embracing deepfakes and AI‐generated images in neuroscience research. *European Journal of Neuroscience*, *58*(3), 2657–2661. https://doi.org/10.1111/ejn.16052 | Neuroscience | Commentary | Deepfakes and AI offer opportunity for experimental research in face perception and visual systems. |
| 11 | Boudníková, O., & Kleisner, K. (2024). AI-generated faces show lower morphological diversity than real faces do. *Anthropological Review*, *87*(1), 81–91. Scopus. https://doi.org/10.18778/1898-6773.87.1.06 | Anthropology | Statistical comparison on real and AI faces | AI faces demonstrate lower morphological diversity compared to real faces. |
| 12 | Bray, S. D., Johnson, S. D., & Kleinberg, B. (2023). Testing human ability to detect ‘deepfake’ images of human faces. *Journal of Cybersecurity*, *9*(1). Scopus. https://doi.org/10.1093/cybsec/tyad011 | Cybersecurity | Experimental survey (Quantitative) | Participants over-estimated confidence in deepfake detection, which was unrelated to detection accuracy. |
| 13 | Cochran, J. D., & Napshin, S. A. (2021). Deepfakes: Awareness, concerns, and platform accountability. *Cyberpsychology, Behavior, and Social Networking*, *24*(3), 164–172. https://doi.org/10.1089/cyber.2020.0100 | Cyberpsychology | Online survey (Quantitative) | People lack confidence in technology to solve the issue of deepfakes. |
| 14 | Cripps, J. (2022). *An intersectional and ecological approach to understanding cyber-sexual violence vulnerability: Connecting individual, environmental, and sociocultural factors* (2022-34091-205; Issues 6-B). ProQuest Information & Learning. | Psychology | Online survey (Quantitative) | Examined social, ecological and intersectional vulnerability factors to cyber-sexual violence victimisation. Engagement in sexting and victimisation history were significant predictors. |
| 15 | Eberl, A., Kühn, J., & Wolbring, T. (2022). Using deepfakes for experiments in the social sciences—A pilot study. *Frontiers in Sociology*, *7*. https://doi.org/10.3389/fsoc.2022.907199 | Sociology | Experimental questionnaire (Quantitative) | Deepfake manipulation of physical attractiveness can influence students’ perceptions of teaching ability. |
| 16 | Fido, D., Rao, J., & Harper, C. A. (2022). Celebrity status, sex, and variation in psychopathy predicts judgements of and proclivity to generate and distribute deepfake pornography. *Computers in Human Behavior*, *129*. https://doi.org/10.1016/j.chb.2021.107141 | Psychology | Online survey (vignette method & quantitative analysis) | People have more lenient judgements of sexualised deepfake images of celebrities, men, and images for self-sexual gratification which are not publicly shared. |
| 17 | Godulla, A., Hoffmann, C. P., & Seibert, D. M. A. (2021). Dealing with deepfakes—An interdisciplinary examination of the state of research and implications for communication studies. *Studies in Communication and Media*, *10*(1), 73–96. Scopus. https://doi.org/10.5771/2192-4007-2021-1-72 | Media Communication | Systematic Review | Real pornographic content is perceptually indistinct from fabricated images or videos. |
| 18 | Goh, D. H.-L. (2024). “He looks very real”: Media, knowledge, and search-based strategies for deepfake identification. *Journal of the Association for Information Science and Technology*, *75*(6), 643–654. Scopus. https://doi.org/10.1002/asi.24867 | Computational social science | Qualitative Interviews | People use both video and audio cues to identify deepfakes. |
| 19 | Gosse, C., & Burkell, J. (2020). Politics and porn: How news media characterizes problems presented by deepfakes. *Critical Studies in Media Communication*, *37*(5), 497–511. Scopus. https://doi.org/10.1080/15295036.2020.1832697 | Media Communication | Discourse analysis of news articles | News media positions deepfake gender-based violence as less important than deepfakes used for disinformation. |
| 20 | Groh, M., Epstein, Z., Firestone, C., & Picard, R. (2022). Deepfake detection by human crowds, machines, and machine-informed crowds. *PNAS Proceedings of the National Academy of Sciences of the United States of America*, *119*(1). https://doi.org/10.1073/pnas.2110013119 | Neuroscience | Experimental questionnaire (Quantitative) | Deepfakes designed to disrupt human visual processing comprise performance on deepfake detection. |
| 21 | Hancock, J. T., & Bailenson, J. N. (2021). The social impact of deepfakes. *Cyberpsychology, Behavior, and Social Networking*, *24*(3), 149–152. Scopus. https://doi.org/10.1089/cyber.2021.29208.jth | Cyberpsychology | Special Editorial | Proposes need for research into social impact of deepfakes. |
| 22 | Harder, S. K. (2023). Re-faced and pornified—A visual, narrative analysis of sexual scripts in police cases of image-based abuse. *The British Journal of Criminology*, *63*(3), 651–667. https://doi.org/10.1093/bjc/azac051 | Criminology | Qualitative analysis of IBSA images and police case reports | Risk of IBSA related to other forms of TFSV such as deepfake manipulation. |
| 23 | Harper, C. A., Fido, D., & Petronzi, D. (2021). Delineating non-consensual sexual image offending: Towards an empirical approach. *Aggression and Violent Behavior*, *58*. https://doi.org/10.1016/j.avb.2021.101547 | Psychology | Literature Review | Distinguished deepfake media production from other forms of IBSA. |
| 24 | Harper, C. A., Smith, L., Leach, J., Daruwala, N. A., & Fido, D. (2023). Development and validation of the beliefs about revenge pornography questionnaire. *Sexual Abuse*, *35*(6), 748–783. Scopus. https://doi.org/10.1177/10790632221082663 | Psychology | Development of questionnaire (Quantitative online survey) | Developed ‘Beliefs about Revenge Pornography Questionnaire’ for future use in psychological research. |
| 25 | Haut, K., Wohn, C., Antony, V., Goldfarb, A., Welsh, M., Sumanthiran, D., Jang, J., Ali, M. R., & Hoque, E. (2021). *Could you become more credible by being White? Assessing Impact of Race on Credibility with Deepfakes* (arXiv:2102.08054). arXiv. https://doi.org/10.48550/arXiv.2102.08054 | Computational social science | Experimental survey (Quantitative) | Manipulation of race on video where person appears whiter, increases perceived credibility of person telling the truth. |
| 26 | Henry, N., & Beard, G. (2024). Image-based sexual abuse perpetration: A scoping review. *Trauma, Violence, and Abuse*. Scopus. https://doi.org/10.1177/15248380241266137 | Psychology | Scoping Review | Lack of prevalence statistics for deepfake perpetration due to integration with other IBSA behaviours. |
| 27 | Henry, N., Flynn, A., & Powell, A. (2020). Technology-facilitated domestic and sexual violence: A review. *Violence Against Women*, *26*(15–16), 1828–1854. Scopus. https://doi.org/10.1177/1077801219875821 | Psychology | Literature Review | Deepfakes can be characterised as using specific AI for digital manipulations to create realistic but false sexualised images, videos, and/or audio. |
| 28 | Huber, A. (2023). ‘A shadow of me old self’: The impact of image-based sexual abuse in a digital society. *International Review of Victimology*, *29*(2), 199–216. https://doi.org/10.1177/02697580211063659 | Social Science | Qualitative Interviews | Social and emotional impacts of deepfake gender-based violence on victim/survivors include reputational harm, relationship difficulties, and physical and mental health challenges such as anxiety and depression. |
| 29 | Jacobs, K. (2024). DIY pornography and the deepfake coup. *Porn Studies*, *11*(1), 91–98. Scopus. https://doi.org/10.1080/23268743.2023.2297691 | Psychology | Literature Review | Frames deepfakes as ‘DIY Porn’, primarily used to target outspoken feminists, LGBTQ+ minorities, and public figures from ethnic minority backgrounds. |
| 30 | Jacobsen, B. N., & Simpson, J. (2024). The tensions of deepfakes. *Information Communication and Society*, *27*(6), 1095–1109. Scopus. https://doi.org/10.1080/1369118X.2023.2234980 | Computational social science | Literature Review | Deepfakes perpetuate objectification of women, represent loss of agency over women’s bodies and a loss of control over images. |
| 31 | Jain, N., Olmo, A., Sengupta, S., Manikonda, L., & Kambhampati, S. (2022). Imperfect ImaGANation: Implications of GANs exacerbating biases on facial data augmentation and snapchat face lenses. *Artificial Intelligence*, *304*. Scopus. https://doi.org/10.1016/j.artint.2021.103652 | Computational social science | Experimental questionnaire and Case Study analysis (Quantitative) | Deep learning models (GANs) produce non-white women with more masculine features and lighter skin when generating images of engineering professors. |
| 32 | Jin, X., Zhang, Z., Gao, B., Gao, S., Zhou, W., Yu, N., & Wang, G. (2023). Assessing the perceived credibility of deepfakes: The impact of system-generated cues and video characteristics. *New Media and Society*. Scopus. https://doi.org/10.1177/14614448231199664 | Media Communication | Experimental questionnaire (Quantitative) | Number of source’s followers, video popularity, length of video and high definition of deepfakes can influence perceived credibility. |
| 33 | Kalpokas, I., Salaseviciute, V., & Lipske, M. (2024). Technology as a threat or a solution? The challenges of responding to synthetic media. *Baltic Journal of Law and Politics*, *16*(2), 1–22. Scopus. https://doi.org/10.2478/bjlp-2023-0010 | Law & Policy | Literature Review | Online communities for sharing non-consensual synthetic sexual imagery display performative hypermasculine violence and toxic masculinity. |
| 34 | Kikerpill, K. (2020). Choose your stars and studs: The rise of deepfake designer porn. *Porn Studies*, *7*(4), 352–356. Scopus. https://doi.org/10.1080/23268743.2020.1765851 | Social Science | Analysis of online Deepfake forums | Exhibited entitlement to create deepfake content of others in community forums online, including ‘designer pornography’ of people known in the past. |
| 35 | Lagrange, B., Van Bauwel, S., Biltereyst, D., Cannizzaro, S., Toms, J., Ağca, Y., Andersson, I., Bjorner, E., Karadimitriou, A., Odstrčilová, K., Papathanassopoulos, S., Risi, E., & Latronico, V. (2024). The future of gender and gender equality online: A scenario analysis of imaginaries on gender and social media platforms. *Central European Journal of Communication*, *17*(1), 103–124. Scopus. https://doi.org/10.51480/1899-5101.17.1(35).704 | Media Communication & Gender Studies | Qualitative analysis of future scenarios | Future of gender equality on social media foreseen as safe spaces for expressions of gender identity and gender activism. |
| 36 | Maddocks, S. (2018). From non-consensual pornography to image-based sexual abuse: Charting the course of a problem with many names. *Australian Feminist Studies*, *33*(97), 345–361. Scopus. https://doi.org/10.1080/08164649.2018.1542592 | Media & Feminist Studies | Qualitative Interviews | Activists advocating for re-naming of ‘revenge pornography’, initiating terminology such as image based sexual abuse (IBSA) and technology-facilitated sexual violence (TFSV), used to inform policy. |
| 37 | Marini, M., Ansani, A., Demichelis, A., Mancini, G., Paglieri, F., & Viola, M. (2024). Real is the new sexy: The influence of perceived realness on self-reported arousal to sexual visual stimuli. *Cognition and Emotion*, *38*(3), 348–360. Scopus. https://doi.org/10.1080/02699931.2023.2296581 | Psychology | Experimental online survey | Perceived realness of images positively correlates to self-reported sexual arousal. |
| 38 | Martínez-Bacaicoa, J., Sorrel, M. A., & Gámez-Guadix, M. (2024). Development and validation of technology-facilitated sexual violence perpetration and victimization scales among adults. *Assessment*. Scopus. https://doi.org/10.1177/10731911241229575 | Psychology | Development of TFSV perpetration and victimisation scale (Quantitative online survey) | Validity of the TFSV scales to be used in future research. |
| 39 | Miller, E. J., Foo, Y. Z., Mewton, P., & Dawel, A. (2023). How do people respond to computer-generated versus human faces? A systematic review and meta-analyses. *Computers in Human Behavior Reports*, *10*. Scopus. https://doi.org/10.1016/j.chbr.2023.100283 | Psychology | Systematic review & meta-analyses | Computer-generated faces can be indistinguishable from human faces, especially in regard to aspects of emotion perception. |
| 40 | Miller, E. J., Steward, B. A., Witkower, Z., Sutherland, C. A. M., Krumhuber, E. G., & Dawel, A. (2023). AI hyperrealism: Why AI faces are perceived as more real than human ones. *Psychological Science*, *34*(12), 1390–1403. Scopus. https://doi.org/10.1177/09567976231207095 | Psychology | Experimental questionnaire | White AI faces perceived as more human than human faces, termed as *AI hyperrealism.* |
| 41 | Misirlis, N., & Munawar, H. B. (2022). From deepfake to deep-useful: risks and opportunities through a systematic literature review. In Kommers P., Sanchez I.A., Isaias P., & Rodrigues L. (Eds.), *Proc. Int. Conf. E-Soc. Mob. Learn.* (pp. 26–32). IADIS Press; Scopus. https://www.scopus.com/inward/record.uri?eid=2-s2.0-85137610892&partnerID=40&md5=17aa0dd8650923c89405ab7b43d4484e | Psychology | Systematic Review | Research interest in deepfake detection algorithms and the use of deepfakes for disinformation. |
| 42 | Monteiro, R., Grangeia, H., & Santos, A. (2024). Technology-facilitated sexual violence: Victimization and risk factors. *Social Sciences*, *13*(7), Article 7. https://doi.org/10.3390/socsci13070372 | Social Science | Online questionnaire | Deepfake prevalence often embedded in IBSA. Gender, age and sexual orientation are significant predictors of TFSV victimisation. |
| 43 | Mumford, E. A., Maitra, P., Sheridan, J., Rothman, E. F., Olsen, E., & Roberts, E. (2023). Technology-facilitated abuse of young adults in the united states: A latent class analysis. *Cyberpsychology*, *17*(3). Scopus. https://doi.org/10.5817/CP2023-3-7 | Cyberpsychology | Development of technology facilitated abuse measures (online survey) | Conflation of deepfakes with other forms of image manipulation, ‘digitally altered images’ included photoshop and deepfake. |
| 44 | Napshin, S., Paul, J., & Cochran, J. (2024). Individual responsibility around Deepfakes: It’s no laughing matter. *Cyberpsychology, Behavior, and Social Networking*, *27*(2), 105–110. https://doi.org/10.1089/cyber.2023.0274 | Cyberpsychology | Online survey | Externalisation of responsibility for sharing deepfake content when difficult to detect, and younger people are more likely to perceive themselves as responsible for sharing humorous deepfake content. |
| 45 | Nas, E., & de Kleijn, R. (2024). Conspiracy thinking and social media use are associated with ability to detect deepfakes. *Telematics and Informatics*, *87*. https://doi.org/10.1016/j.tele.2023.102093 | Psychology | Experimental online survey | Higher deepfake detection performance is associated with familiarity of person in image, more time spent on social media, and conspiracy thinking. |
| 46 | Newton, O. B., & Stanfill, M. (2020). My NSFW video has partial occlusion: Deepfakes and the technological production of non-consensual pornography. *Porn Studies*, *7*(4), 398–414. Scopus. https://doi.org/10.1080/23268743.2019.1675091 | Psychology | Analysis of deepfake communities (Quantitative and Qualitative) | Deepfake communities reflect a culture of toxic ‘geek’ masculinity. |
| 47 | Nightingale, S. J., & Wade, K. A. (2022). Identifying and minimising the impact of fake visual media: Current and future directions. *Memory, Mind and Media*, *1*. Scopus. https://doi.org/10.1017/mem.2022.8 | Psychology | Literature Review | Importance for future research to examine ability for deepfake detection. |
| 48 | Öhman, C. (2022). The identification game: Deepfakes and the epistemic limits of identity. *Synthese*, *200*(4). Scopus. https://doi.org/10.1007/s11229-022-03798-5 | Philosophy | Essay | Difficulty in identifying personal distinguishing features such as face and voice in synthetic media and deepfakes. |
| 49 | Paradiso, M. N., Rollè, L., & Trombetta, T. (2024). Image-based sexual abuse associated factors: A systematic review. *Journal of Family Violence*, *39*(5), 931–954. Scopus. https://doi.org/10.1007/s10896-023-00557-z | Psychology | Systematic Review | Recognition of deepfake as a distinct form of IBSA. |
| 50 | Plohl, N., Mlakar, I., Aquilino, L., Bisconti, P., & Smrke, U. (2024). Development and validation of the perceived deepfake trustworthiness questionnaire (PDTQ) in three languages. *International Journal of Human-Computer Interaction*. Scopus. https://doi.org/10.1080/10447318.2024.2384821 | Psychology | Development of Perceived Deepfake Trustworthiness Questionnaire (PDTQ) (Qualitative Interviews and Quantitative surveys) | Validity of Deepfake Trustworthiness Questionnaire (PDTQ) for use in future deepfake research. |
| 51 | Popova, M. (2020). Reading out of context: Pornographic deepfakes, celebrity and intimacy. *Porn Studies*, *7*(4), 367–381. Scopus. https://doi.org/10.1080/23268743.2019.1675090 | Social Science | Qualitative analysis of deepfake websites | Deepfake sexualised content of celebrities on dedicated websites exhibit practices of community-building and shared fan-fiction. |
| 52 | Rackley, E., McGlynn, C., Johnson, K., Henry, N., Gavey, N., Flynn, A., & Powell, A. (2021). Seeking justice and redress for victim-survivors of image-based sexual abuse. *Feminist Legal Studies*, *29*(3), 293–322. Scopus. https://doi.org/10.1007/s10691-021-09460-8 | Feminist Legal Studies | Quantitative surveys and Qualitative interviews | Examined prevalence rates of IBSA, 34% of cases involved digitally altered images, which included ‘fakeporn’ and photoshopped images. |
| 53 | Sambasivan, N., Batool, A., Ahmed, N., Matthews, T., Thomas, K., Gaytán-Lugo, L. S., Nemer, D., Bursztein, E., Churchill, E., & Consolvo, S. (2019). ‘They don’t leave us alone anywhere we go’: Gender and digital abuse in south Asia. *Proceedings of the 2019 CHI Conference on Human Factors in Computing Systems*, 1–14. https://doi.org/10.1145/3290605.3300232 | Feminist & Cyber psychology | Qualitative Interviews and Focus groups | 6% of women in South Asian countries had been victimised by synthetic porn, which included all forms of superimposing images (not exclusively deepfake). Coping strategies for women against digital abuse include removing themselves from profile pictures. |
| 54 | Sanders, T., Trueman, G., Worthington, K., & Keighley, R. (2023). Non-consensual sharing of images: Commercial content creators, sexual content creation platforms and the lack of protection. *New Media and Society*. Scopus. https://doi.org/10.1177/14614448231172711 | Sociology | Mixed methods (Qualitative Interviews and Quantitative online survey) | Lack of deepfake specificity when assessing prevalence of misused content of commercial content creators. |
| 55 | Sen, R., & Jha, S. (2024). Women under Hindutva: Misogynist memes, mock-auction and doxing, deepfake-pornification and rape threats in digital space. *Journal of Asian and African Studies*. Scopus. https://doi.org/10.1177/00219096241257686 | Gender & Feminist Studies | Analysis of case studies | Deepfake abuse, deepfake pornification and sexually manipulated images are used in India to mute women in cyberspace who defy cultural and religious belief systems. |
| 56 | Sheikh, M. M. R., & Rogers, M. M. (2024). Technology-facilitated sexual violence and abuse in low and middle-income countries: A scoping review. *Trauma, Violence, & Abuse*, *25*(2), 1614–1629. https://doi.org/10.1177/15248380231191189 | Sociology | Scoping Review | Reported on Sambasivan et al. (2019) findings indicating prevalence rates of deepfake harassment, which also encompassed photoshopped images. |
| 57 | Somoray, K., & Miller, D. J. (2023). Providing detection strategies to improve human detection of deepfakes: An experimental study. *Computers in Human Behavior*, *149*, 1–8. https://doi.org/10.1016/j.chb.2023.107917 | Psychology | Experimental online survey | People overestimate their pre-emptive abilities to detect deepfakes. |
| 58 | Tanwar, R., & Mahapatra, D. M. (2024). Decoding digital deception: Exploring motivations in online gaming bullying, cyber frauds and deep fake AI. *International Journal of Interdisciplinary Approaches in Psychology*, *2*(5), 1847-1865. | Psychology | Qualitative analysis of interviews, online forums and case studies | Deepfake creation is a multi-faceted, highly complex phenomenon with a range of psychological drivers. |
| 59 | Turner, H. A., Finkelhor, D., & Colburn, D. (2024). Contexts and characteristics of imaged-based sexual exploitation and abuse of children: Incident dynamics in a national sample. *Child Maltreatment*. Scopus. https://doi.org/10.1177/10775595241233970 | Sociology | Online survey | Conflation of deepfake terminology with photoshopping behaviour when assessing prevalence statistics. |
| 60 | van der Nagel, E. (2020). Verifying images: Deepfakes, control, and consent. *Porn Studies*, *7*(4), 424–429. Scopus. https://doi.org/10.1080/23268743.2020.1741434 | Media Studies | Analysis of case studies | Image verification system on Reddit for deepfakes allows women to reclaim agency over their images. |
| 61 | Vasist, P. N., & Krishnan, S. (2023). Engaging with deepfakes: A meta-synthesis from the perspective of social shaping of technology theory. *Internet Research*, *33*(5), 1670–1726. Scopus. https://doi.org/10.1108/INTR-06-2022-0465 | Sociology | Meta-synthesis of qualitative literature | Creation of deepfakes requires technological expertise, therefore examining motivation in isolation doesn’t provide a comprehensive view of deepfake perpetration. |
| 62 | Wagner, T. L., & Blewer, A. (2019). ‘The word real is no longer real’: Deepfakes, gender, and the challenges of AI-altered video. *Open Information Science*, *3*(1), 32–46. Scopus. https://doi.org/10.1515/opis-2019-0003 | Gender & Feminist Studies | Literature Review | Need for research to examine deepfakes and artificial intelligence with feminist perspective to acknowledge gender disparities in visual information. |
| 63 | Wang, S., & Kim, S. (2022). Users’ emotional and behavioral responses to deepfake videos of K-pop idols. *Computers in Human Behavior*, *134*. https://doi.org/10.1016/j.chb.2022.107305 | Psychology & Media Communication | Online Survey | Anger more strongly associated with bystander intervention than guilt when exposed to sexualised deepfake content. |
| 64 | Wu, F., Ma, Y., & Zhang, Z. (2021). ‘I found a more attractive deepfaked self’: The self-enhancement effect in deepfake video exposure. *Cyberpsychology, Behavior, and Social Networking*, *24*(3), 173–181. https://doi.org/10.1089/cyber.2020.0173 | Cyberpsychology | Experimental online questionnaire | Women’s own use of deepfake can positively mediate self-perceived attractiveness. |
